# The Rcs System in *Enterobacteriaceae*: Envelope Stress Responses and Virulence Regulation

**DOI:** 10.3389/fmicb.2021.627104

**Published:** 2021-02-15

**Authors:** Jiao Meng, Glenn Young, Jingyu Chen

**Affiliations:** ^1^Beijing Laboratory for Food Quality and Safety, College of Food Science and Nutritional Engineering, China Agricultural University, Beijing, China; ^2^Department of Food Science and Technology, University of California, Davis, Davis, CA, United States

**Keywords:** Rcs system, envelope stress response, virulence regulation, environmental adaptation, *Enterobacteriaceae*

## Abstract

The bacterial cell envelope is a protective barrier at the frontline of bacterial interaction with the environment, and its integrity is regulated by various stress response systems. The Rcs (regulator of capsule synthesis) system, a non-orthodox two-component regulatory system (TCS) found in many members of the *Enterobacteriaceae* family, is one of the envelope stress response pathways. The Rcs system can sense envelope damage or defects and regulate the transcriptome to counteract stress, which is particularly important for the survival and virulence of pathogenic bacteria. In this review, we summarize the roles of the Rcs system in envelope stress responses (ESRs) and virulence regulation. We discuss the environmental and intrinsic sources of envelope stress that cause activation of the Rcs system with an emphasis on the role of RcsF in detection of envelope stress and signal transduction. Finally, the different regulation mechanisms governing the Rcs system’s control of virulence in several common pathogens are introduced. This review highlights the important role of the Rcs system in the environmental adaptation of bacteria and provides a theoretical basis for the development of new strategies for control, prevention, and treatment of bacterial infections.

## Envelope Stress Responses

**FIGURE 1:**
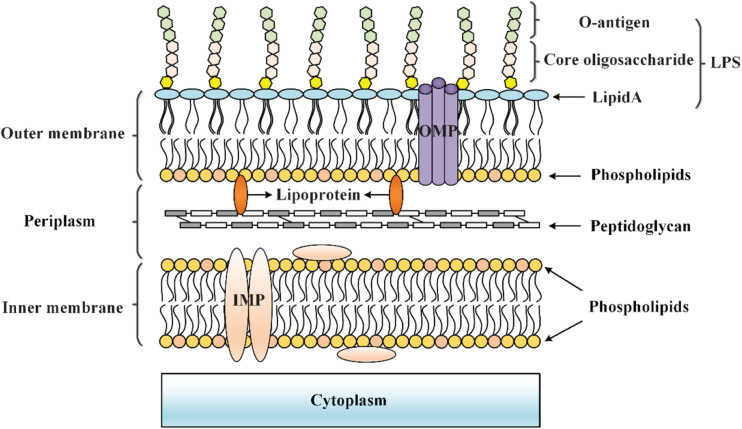
Cell envelope structure of Gram-negative bacteria. The cell envelope of Gram-negative bacteria is composed of an OM that is separated from the IM by an aqueous periplasmic space that houses the peptidoglycan cell wall, thereby acting as a protective barrier at the frontline of the interaction between bacteria and the environment.

The cell envelope of Gram-negative bacteria is generally composed of an inner membrane (IM), a periplasm with a thin peptidoglycan layer, and an outer membrane (OM) (Grabowicz and Silhavy, 2017) (Figure 1). The OM is a permeable barrier involved in the exchange of substances between the cell and the environment (Nikaido, 1989, 2003). The OM is an asymmetric lipid bilayer in which lipopolysaccharides (LPS), composed of lipid A, core oligosaccharide, and O-antigen, form surface-exposed leaflet, while phospholipids form internal leaflet. Among them, saturated acyl chains and hydrophilic lateral interactions between LPS bound by divalent cations hinder the ability of large hydrophilic and hydrophobic molecules to penetrate the OM (Raetz and Whitfield, 2002). Small molecular nutrients (<600 Daltons) can pass through the OM through the outer membrane β-barrel proteins (OMPs) called porins (O’Shea and Moser, 2008; Li X. Z. et al., 2015). Additionally, the periplasm exerts a remarkable protective effect as it contains many molecules related to the protection of cells from stress, as well as key proteins related to transportation and metabolism (Mullineaux et al., 2006). More importantly, peptidoglycan in the periplasmic space and OM can create a load-bearing structure in bacteria, thereby allowing them to resist mechanical stress and osmotic stress (Rojas et al., 2018). The IM is the location of key cellular functions, in which inner membrane proteins (IMPs) use different pathways for membrane targeting and integration. Generally, the IM is the ultimate barrier between the environment and the cytoplasm (Silhavy et al., 2010). Toxic molecules are prevented from accumulating within cells by efflux pumps that span the cell envelope (Nagakubo et al., 2002; Li X. Z. et al., 2015). In summary, the cell envelope is a protective barrier involved in the interaction between bacteria and the environment, and it provides bacteria with considerable resistance to environmental damage and toxic molecules.

Bacteria are exposed to various envelope stresses in both free-living environmental and infectious lifestyles (Grabowicz and Silhavy, 2017). Cell envelope stress may be due to environmental flux (such as increased osmolarity, redox stress, and exposure to toxic molecules and antimicrobials) and intrinsic stress sources (such as errors in biosynthesis, assembly and transport of membrane components, protein misfolding, and mutations) (Mitchell and Silhavy, 2019). Indeed, the envelope must be repaired and modified in response to these stresses. Bacterial envelope stress responses (ESRs) sense cell envelope damage or defects and alter the transcriptome to relieve destructive stress, which is particularly important for survival and virulence regulation (Grabowicz and Silhavy, 2017; Mitchell and Silhavy, 2019). Presently, multiple envelope stress response pathways, including σ^E^ response system, Cpx response system, Bae response system, Psp response system, and regulator of capsule synthesis (Rcs) response system are known to sense and respond to cell envelope assaults (Mitchell and Silhavy, 2019) (Table 1). The σ^E^ system can respond to unfolded OMPs in the periplasm. After the σ^E^ system is activated, genes are expressed from σ^E^-dependent promoters, leading to the upregulation of the OMP folding pathway (Mecsas et al., 1993; Walsh et al., 2003; Rhodius et al., 2006). The Cpx system is induced by defects in IM protein secretion or by misfolding of IM or periplasmic proteins that may occur due to a variety of situations, including changes in pH or osmolarity, cell adherence to the hydrophobic surfaces, peptidoglycan biosynthesis defects, and copper exposure. The functional result of Cpx activation is the direct or indirect transcriptional repression of genes encoding proteins that form non-essential IM protein complexes and the increased expression of genes related to peptidoglycan modification, efflux, and metal and redox homeostasis (Danese and Silhavy, 1998; Otto and Silhavy, 2002; Jubelin et al., 2005; Yamamoto and Ishihama, 2006; Evans et al., 2013; López et al., 2018; Delhaye et al., 2019; May et al., 2019). The Bae system is activated by exposure to toxic molecules including ethanol, indole, nickel chloride, sodium tungstate, and zinc, which leads to the upregulation of genes encoding periplasmic chaperones and efflux pumps (Nagakubo et al., 2002; Raffa and Raivio, 2002; Zhou et al., 2003; Nishino et al., 2005; Bury-Moné et al., 2009; Leblanc et al., 2011). The Psp system is induced by severe damage to the IM, including infection by filamentous phages, extreme heat shock, osmotic shock, organic solvent exposure, disruption of protein secretion, and localization of OMPs at the IM, which then increases the transcription of *psp* genes to counter these stresses (Brissette et al., 1990; Carlson and Silhavy, 1993; Jovanovic et al., 1996; Kobayashi et al., 1998; Jones et al., 2003). The Rcs system is activated by OM damage, LPS synthesis defects, peptidoglycan perturbation, and lipoprotein mislocalization (specific input signals are shown in Table 2), which then causes changes in the expression of genes involved in capsule biosynthesis, motility, biofilm formation, and virulence (Girgis et al., 2007; Callewaert et al., 2009; Farris et al., 2010; Tao et al., 2012; Konovalova et al., 2016; Meng et al., 2020a).

**TABLE 1:** Overview of envelope stress response pathways.

Pathways	Envelope stresses	Activation mechanisms	Targets	References
σ^E^ system	Unfolded OMPs	The unfolded OMP in the periplasm binds to the IM protease DegS, causing the conformational change of DegS and enabling it to cleave anti-sigma factor RseA, thereby removing the inhibitory effect of RseA on the σ^E^ system	OMP folding pathways	Mecsas et al., 1993; Walsh et al., 2003; Rhodius et al., 2006
Cpx system	Defects in IM protein secretion; misfolding of IM or periplasmic protein; lipoprotein export defect	Upon receiving a stimulus, CpxA autophosphorylates at the histidine kinase (HK) domain, and the phosphoryl group is transferred to the phosphoryl receiver (PR) domain of response regulator CpxR to activate the Cpx system	IM protein complexes; protein folding and degradation; peptidoglycan modification; efflux; metal and redox homeostasis	Danese and Silhavy, 1998; Otto and Silhavy, 2002; Jubelin et al., 2005; Yamamoto and Ishihama, 2006; Evans et al., 2013; López et al., 2018; Delhaye et al., 2019; May et al., 2019
Bae system	Toxic molecules	Upon receiving a stimulus, the BaeS autophosphorylates at its HK domain, and the phosphoryl group is transferred to the PR domain of the response regulator BaeR to activate the Bae system	Periplasmic chaperone; efflux	Nagakubo et al., 2002; Raffa and Raivio, 2002; Zhou et al., 2003; Nishino et al., 2005; Bury-Moné et al., 2009; Leblanc et al., 2011
Psp system	Severe damage to the IM	Upon receiving a stimulus, the IM proteins PspB and PspC interact with PspA, which releases PspF to activate the Psp system	*psp* genes	Brissette et al., 1990; Carlson and Silhavy, 1993; Jovanovic et al., 1996; Kobayashi et al., 1998; Jones et al., 2003
Rcs system	OM damage; LPS synthesis defects; peptidoglycan perturbation; lipoprotein mislocalization	Upon receiving a stimulus, RcsC autophosphorylates at its HK domain. The phosphoryl group is finally transferred to the PR domain of response regulator RcsB through multiple transfer steps to activate the Rcs system	Capsule; motility; biofilm formation; virulence	Girgis et al., 2007; Callewaert et al., 2009; Farris et al., 2010; Tao et al., 2012; Konovalova et al., 2016; Meng et al., 2020a

**TABLE 2:** Examples of envelope stresses and effects that induce the Rcs system.

Sources	Input signals	Species	Envelope effects	References
Environmental stress	Increased osmolarity	*E. coli*	Perturbation of the membrane tension	Sledjeski and Gottesman, 1996
	Lysozyme	*E. coli*	Destruction of the periplasmic peptidoglycan	Callewaert et al., 2009
	Mecillinam	*E. coli*	Destruction of the periplasmic peptidoglycan	Laubacher and Ades, 2008
	β-lactam antibiotics	*E. coli*	Inhibition of periplasmic peptidoglycan formation	Hirakawa et al., 2003
	Redox stress	*S. enterica* serovar Typhimurium	Oxidative damage to membranes and proteins	Farizano et al., 2014
	Cationic antimicrobial peptides	*S. enterica*	OM damage	Farris et al., 2010
Intrinsic stress	*waaF* deletion	*E. coli*	Defect in LPS synthesis	Ren et al., 2016
	*ugd deletion*	*Ed. tarda*	Defect in LPS synthesis	Lv et al., 2012
	*lolA* mutation	*E. coli*	Lipoprotein mislocalization	Tao et al., 2012
	*tolA* deletion	*E. coli*	OM perturbation	Morgan et al., 2014
	*bamA* promoter (*bamA*101) mutation	*E. coli*	Defect in occluding RcsF from IgaA	Cho et al., 2014
	*ompA* deletion	*E. coli*	Defect in occluding RcsF from IgaA	Cho et al., 2014
	*dsbA* deletion	*S. enterica*	Defect in RcsF disulfide bonds	Lin et al., 2008
	*pbp4*, *pbp5*, *pbp7* or *ampH* deletion	*E. coli*	Modification of the periplasmic peptidoglycan	Evans et al., 2013
	*opgGH* deletion	*Y. enterocolitica*	Mutation in periplasmic glycans that affect peptidoglycan	Meng et al., 2020a
	*mdoH* deletion	*E. coli*	Mutation in periplasmic glycans that affect peptidoglycan	Shiba et al., 2012

Among these stress response pathways, the Rcs pathway is intriguingly unique due to the following features: (i) the Rcs system is the only signal transduction system known to have an OM component (RcsF) that senses almost all induction cues (Gervais and Drapeau, 1992; Majdalani and Gottesman, 2005; Cho et al., 2014); and (ii) the response regulator RcsB can form homodimers or heterodimers with a variety of auxiliary proteins to regulate its target genes, thereby conferring flexibility to the Rcs system and allowing bacteria to be fine-tuned to complex environments (Stout et al., 1991; Castanié-Cornet et al., 2010; Venkatesh et al., 2010; Pannen et al., 2016). Numerous studies have shown that the Rcs system plays an important role in sensing envelope stress and regulating the physiological behavior of bacteria, especially virulence, thereby enabling bacteria to better adapt to environmental changes (Hirakawa et al., 2003; Erickson and Detweiler, 2006; Farizano et al., 2014; Meng et al., 2020b). Therefore, this review focuses on the important roles of the Rcs system in ESRs and bacterial virulence regulation.

## An Overview of the Rcs System

### The Components of the Rcs System

The Rcs system was first identified in 1985 as a positive regulator of the biosynthesis of the capsular polysaccharide colanic acid of *Escherichia coli* (Gottesman et al., 1985). Subsequent studies have shown that the Rcs system is a non-orthodox two-component regulatory system (TCS) present in many members of the *Enterobacteriaceae* family of Gram-negative bacteria (Gottesman et al., 1985; Majdalani and Gottesman, 2005; Guo and Sun, 2017). The Rcs system consists of three core proteins, namely the transmembrane hybrid kinase RcsC, the transmembrane protein RcsD, and the response regulator RcsB. The RcsC has both kinase and phosphatase activities, and together with the response regulator RcsB, represents the classic members of bacterial TCS, while the RcsD lacks kinase activity (Majdalani and Gottesman, 2005). A recent study showed that the periplasmic domain of RcsC is dispensable for sensing the inducing signals, and the Rcs activity is not regulated at the level of RcsC (Wall et al., 2020). In the absence of any environmental signal, RcsC and RcsD together act as phosphatases to ensure that phosphorylated RcsB (RcsB-P) in the cell is maintained at a low level (Clarke, 2010). RcsF is an OM lipoprotein required for the perception of several envelope stress signals that have been shown to activate the Rcs system (Gervais and Drapeau, 1992; Majdalani and Gottesman, 2005; Castanie-Cornet et al., 2006). IgaA (YrfF in *Salmonella enterica* serovar Typhimurium) is an IM protein that seems to function by inhibiting the Rcs signaling, thus ensuring that the signal through this phosphorylation is minimal in the absence of environmental stimuli (Dominguez-Bernal et al., 2004; Hussein et al., 2018; Wall et al., 2020). RcsA is an auxiliary protein that assists RcsB binding to the sites marked as RcsAB boxes (Pristovsek et al., 2003). In addition to RcsA, many auxiliary proteins such as BglJ, MatA (EcpR), and GadE were shown to interact with RcsB (Castanié-Cornet et al., 2010; Venkatesh et al., 2010; Pannen et al., 2016).

### Phosphorylation of the Rcs System

**FIGURE 2:**
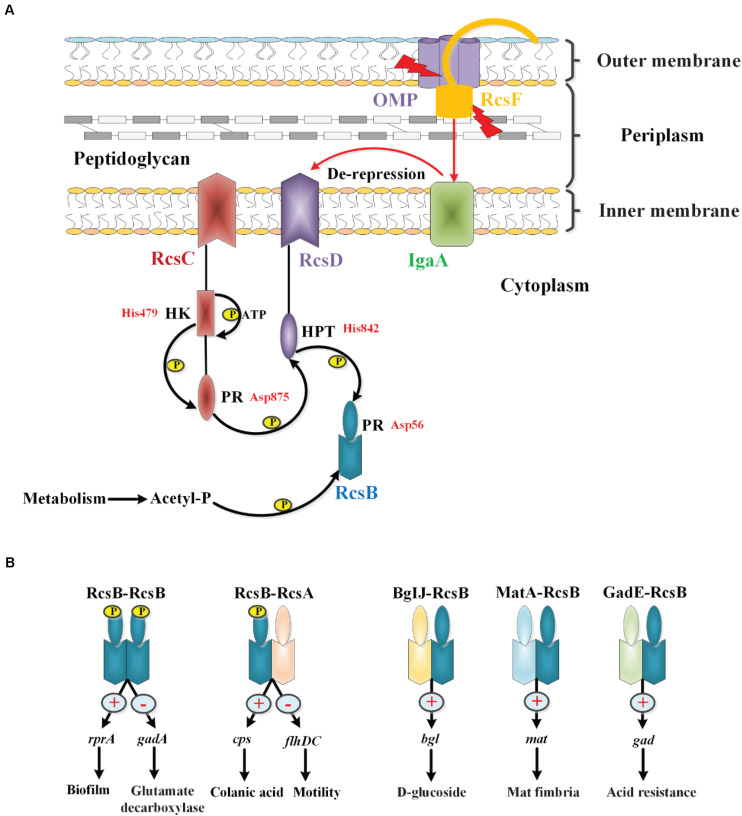
The phosphorylation and regulation of the Rcs system in *E. coli*. **(A)** The core components of the Rcs phosphorelay and the transfer of phosphate. RcsF, the OM lipoprotein that senses signals from the OM and periplasm, is seated in an OMP within the OM and is shown interacting with the periplasmic domain of IgaA. IgaA, a five-pass IM protein, is a negative regulator of the phosphorelay. Current model suggests that upon stress signaling, RcsF increases or changes contacts with IgaA, leading to de-repression of the phosphorelay. Then the RcsC autophosphorylates at the HK domain in an ATP-dependent manner. The phosphoryl group is then transferred to the PR domain of RcsC, to the HPT domain of RcsD, and finally to RcsB in a successive manner. In the absence of stress, acetyl phosphate may act as a phosphoryl group donor to maintain a low level of RcsB-P. **(B)** Regulation of RcsB homodimers and heterodimers. RcsB can form an RcsB-RcsB homodimer or an RcsB-RcsA heterodimer in an RcsB phosphorylation-dependent manner, or form BglJ-RcsB, MatA-RcsB, and GadE-RcsB heterodimers in an RcsB phosphorylation-independent manner, which then interact with a conserved motif in target genes to modulate their transcription, thereby regulating the physiological activities of bacteria.

The phosphoryl transfer steps of the Rcs system generally follow the His-Asp-His-Asp pathway. According to the system observed in *E. coli*, upon receiving an extracytoplasmic stimulus, likely via RcsF, the hybrid sensor RcsC autophosphorylates at the conserved histidine residue His479 on its histidine kinase (HK) domain in an ATP-dependent manner. The phosphoryl group is then transferred to the aspartate residue Asp875 on the phosphoryl receiver (PR) domain of RcsC. The phosphoryl group is subsequently transferred to the histidine residue His842 on the histidine-containing phosphotransmitter (HPT) domain of RcsD, and finally to the aspartate residue Asp56 on the PR domain of RcsB (Chen et al., 2001; Takeda et al., 2001; Clarke et al., 2002; Majdalani and Gottesman, 2005). Additionally, in the absence of external stimuli or in response to certain metabolic stresses, RcsB can also be phosphorylated by low-molecular-weight phosphodonors such as acetyl phosphate (AcP) (Hu et al., 2013). Recently, Wall et al. have found that IgaA interacts with the phosphorelay protein RcsD, and the interactions between IgaA and RcsD within their respective periplasmic domains of these two proteins anchor repression of signaling. However, the signaling response depends on a second interaction between a truncated Per-Arndt-Sim (PAS-like) domain in RcsD and cytoplasmic loop 1 of IgaA. In their model, the change in the IgaA-RcsD interaction allows RcsC-generated phosphate to flow from RcsC to RcsD, and then to RcsB, activating RcsB-dependent transcription (Wall et al., 2020). However, whether IgaA interacts with RcsC remains to be clarified. So while the phosphate flow is from RcsC to RcsD and then to RcsB, the signaling cascade comes from RcsF or RcsF independent to IgaA to RcsD, which then activates the autophosphorylation of RcsC to start the phosphate flow. Our current understanding of the Rcs phosphorelay is shown in Figure 2A. In addition, the interaction of RcsC and RcsF in the periplasm may be involved in signal transduction in the Rcs system (Sato et al., 2017).

### Regulation of RcsB Homodimers and Heterodimers

RcsB can form homodimers or heterodimers with auxiliary proteins such as RcsA, BglJ, MatA, and GadE, and then bind to a conserved motif in Rcs-regulated genes to activate or inhibit transcription. For example, the RcsB-RcsB homodimer positively regulates sRNA *rprA* and negatively regulates *gadA* in an RcsB phosphorylation-dependent manner to modulate biofilm formation and glutamate decarboxylase synthesis, respectively, in *E. coli* (Majdalani et al., 2002; Castanié-Cornet et al., 2010). The RcsB-RcsA heterodimer activates the expression of the operon and represses expression of the *flhDC* operon to regulate the capsular polysaccharide colanic acid and flagella synthesis, respectively, in an RcsB-phosphorylation-dependent manner in *E. coli* (Stout et al., 1991; Francez-Charlot et al., 2003). It has been reported that RcsA function depends on RcsB phosphorylation, while the effects of other auxiliary regulators, such as BglJ, MatA, and GadE, function independently of phosphorylation of RcsB (Figure 2B). In *E. coli*, the BglJ-RcsB heterodimer activates the *bgl* gene involved in D-glucoside synthesis (Venkatesh et al., 2010); the MatA-RcsB heterodimer activates the *mat* operon involved in Mat fimbria biosynthesis (Pannen et al., 2016); the GadE-RcsB heterodimer activates *gad* genes related to acid resistance (Castanié-Cornet et al., 2010). In general, the participation of multiple auxiliary proteins confers flexibility to the Rcs system, thereby allowing for its adaptation to be fine-tuned to complex environments.

### Sources of Envelope Stresses for Induction of the Rcs System

#### Rcs Activation via Environmental Stress

The osmotic upshift was the first reported environmental signal that could activate the Rcs system in *E. coli* (Sledjeski and Gottesman, 1996), and other input signals involved in envelope stress have been discovered since then (Table 2). One of the main functions of the Rcs system in response to envelope stress is to protect cells from environmental challenges (Mitchell and Silhavy, 2019). For example, when the Rcs system is activated by osmotic stress, the *cps* operon is transiently expressed, thereby allowing the cell to produce colanic acid to enable cellular response to environmental stress that is potentially lethal to bacteria (Alon, 2007). Lysozyme destroys the periplasmic peptidoglycan and induces activation of the Rcs system, which upregulates the transcription of lysozyme inhibitors in *E. coli*, thereby reducing the damage of lysozyme to cells (Callewaert et al., 2009). In addition to lysozyme, Mecillinam can also affect peptidoglycan, thereby inducing the Rcs system (Laubacher and Ades, 2008). Oxidative stress in *S. enterica* serovar Typhimurium can cause damage to the OM and activate the Rcs system, which regulates the transcription of *dps* genes, thereby protecting bacterial DNA from host reactive oxygen species (ROS)-mediated damage during infection (Farizano et al., 2014). The cell membrane damage induced by cationic antimicrobial peptides such as polymyxin B in *S. enterica* can also lead to the activation of the Rcs system. PMB induction at sublethal levels is transient, which indicates that activation of the Rcs system may induce cell surface modification, thereby reducing cationic peptide damage to the OM (Farris et al., 2010). In addition to these environmental stresses, the inhibition of peptidoglycan formation by repression of the penicillin-binding protein (PBPs) caused by β-lactam antibiotics is also an important input signal for activation of the Rcs system (Hirakawa et al., 2003).

#### Rcs Activation via Intrinsic Sources of Stress

Toxic substances produced by cell metabolism, translation stress caused by lack of specific amino acids, and mutations that cause biogenesis alteration can all be considered as intrinsic sources of envelope stress (Mitchell and Silhavy, 2019). Mutations that cause alterations in envelope biogenesis pathways are the main Rcs input signals that are often studied in cases of intrinsic stress (Guo and Sun, 2017) (Table 2). In *E. coli*, these signals include LPS core sugar deficiency caused by *waaF* deletion (Ren et al., 2016), lipoprotein mislocalization caused by *lolA* mutation (Tao et al., 2012), OM perturbation caused by *tolA* deletion (Morgan et al., 2014), modification of the peptidoglycan caused by *pbp4*, *pbp5*, *pbp7*, or *ampH* deletion (Evans et al., 2013), defect in occluding RcsF from IgaA caused by *bamA* promoter (*bamA*101) mutation and *ompA* deletion (Cho et al., 2014). Mutations in periplasmic glycans (formed by enzymes encoded by *mdoG* and *mdoH*, now referred to as *opgG* and *opgH*) can affect peptidoglycans, thereby inducing the Rcs system. For instance, osmoregulated periplasmic glucans (OPGs) defects caused by *opgGH* deletion in *Yersinia enterocolitica* (Meng et al., 2020a) or membrane-derived oligosaccharides synthesis defects caused by *mdoH* deletion in *E. coli* (Shiba et al., 2012) both lead to the activation of the Rcs system. Additionally, defects in LPS (a truncated core with no O-antigen attached) caused by *ugd* deletion in *Edwardsiella tarda* can also modulate the activity of the Rcs system (Lv et al., 2012).

In summary, the perturbation in the cell surface (OM/LPS), periplasmic signals that perturb peptidoglycan, and lipoprotein mislocalization caused by environmental stress or intrinsic sources of stress lead to the activation of the Rcs system, which in turn regulates its target genes to allow cells to adapt to environmental and genetic changes.

### The Role of RcsF in Detection of Envelope Stress

#### RcsF Is Necessary for Sensing Most Induction Signals

RcsF is an OM lipoprotein that contains a lipidated N-terminal membrane-anchored helix followed by a 30-amino acid proline-rich linker and a well-folded 87-amino acid periplasmic domain (Leverrier et al., 2011; Rogov et al., 2011; Umekawa et al., 2013). RcsF localization to the OM requires the Lol system (Konovalova and Silhavy, 2015). The periplasmic domain of RcsF contains four conserved cysteines. Studies have shown that disruption of RcsF disulfide bonds (such as by generation of mutations in DsbA and DsbC) prevents the activation of the Rcs phosphorelay by signals that function through RcsF (Kadokura et al., 2004; Leverrier et al., 2011; Rogov et al., 2011). RcsF overexpression activates the Rcs system, suggesting that increased RcsF levels help to activate the phosphorelay, although there is no evidence that normal induction signals act by increasing RcsF levels (Gervais and Drapeau, 1992). RcsF senses cell envelope stress from the OM and periplasm, and then transmits the signal to the downstream components of the Rcs system. Finally, the Rcs system is activated in response to OM or peptidoglycan damage (Girgis et al., 2007; Callewaert et al., 2009; Farris et al., 2010; Tao et al., 2012; Konovalova et al., 2016; Meng et al., 2020c).

#### The Interaction of RcsF and IgaA to Control the Switch of the Rcs System

IgaA is a five-pass transmembrane protein, which was first identified in *S. enterica* for its effect on intracellular growth and virulence (Cano et al., 2001). The *igaA* gene is also found in other species of *Enterobacterales* that encode the Rcs system (Clarke, 2010). As mentioned above, the IM protein IgaA is an essential negative regulator of Rcs signaling (Dominguez-Bernal et al., 2004; Hussein et al., 2018; Wall et al., 2020). Ample evidence suggests that RcsF does not directly transmit the stress signal from the envelope to the downstream components of the Rcs system, RcsC, RcsD, and RcsB, but through the interaction with IgaA to counteract its negative regulatory effect on Rcs signaling (Cho et al., 2014; Wall et al., 2018). When RcsF interacts with IgaA, the Rcs system is activated (Hussein et al., 2018).

#### RcsF-Dependent Signal Transduction

**FIGURE 3:**
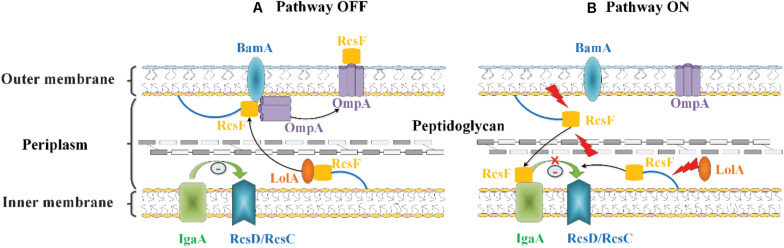
RcsF-dependent signal transduction of the Rcs system in *E. coli*. RcsF, as an OM lipoprotein, is transported to the inner leaflet of the OM by the chaperone LolA. **(A)** Under an unstressed condition, RcsF binds to BamA, which assembles RcsF and OmpA together as a complex. The complex displays RcsF on the cell surface, thereby occluding RcsF from IgaA, an IM protein which inhibits the activation of Rcs signaling. **(B)** Upon stress occurrence in the OM or peptidoglycan, the transported RcsF cannot interact with BamA. Then, the RcsF exposed to the periplasm binds to IgaA and inhibits its function, resulting in the activation of the Rcs system. Additionally, when the LolA is destroyed, newly synthesized RcsF interacts with IgaA due to a failure in transportation to the OM.

RcsF at the OM is surface exposed within the lumen of OMPs. A recent study showed that OmpA is unlikely the vehicle allowing RcsF to reach the surface (Dekoninck et al., 2020). Components of the Bam machinery, which assemble and localize OMPs, are needed to localize RcsF within the OMPs (Cho et al., 2014; Konovalova et al., 2014, 2016; Dekoninck et al., 2020; Rodríguez-Alonso et al., 2020). Rodríguez-Alonso et al. reported the crystal structure of the key BAM component BamA in complex with RcsF and revealed how BamA interacts with RcsF. This finding provided insights into the mechanism used by BAM to assemble RcsF-OMP complexes, a new activity by which BAM exports this lipoprotein to the cell surface (Rodríguez-Alonso et al., 2020). So far, there are two models that reveal the mechanism by which envelope stress induces the Rcs system via RcsF, which were proposed by Silhavy’s lab and Collet’s lab, respectively (Cho et al., 2014; Konovalova et al., 2016). Cho et al. proposed that the interaction of RcsF with BamA and three β-barrels (OmpA, OmpF, and OmpC) plays a major role in RcsF sensing (Cho et al., 2014). In the absence of envelope stress, BamA continuously funnels RcsF through the β-barrel OmpA, and displays RcsF on the cell surface. In this case, RcsF did not interact with IgaA to activate the Rcs system (Cho et al., 2014) (Figure 3A). In the presence of envelope stress, BamA failed to bind RcsF and funnel it to OmpA. In this situation, RcsF interacts with IgaA, which releases the inhibition of IgaA to Rcs signaling, which then leads to the activation of the Rcs system (Cho et al., 2014) (Figure 3B).

**FIGURE 4:**
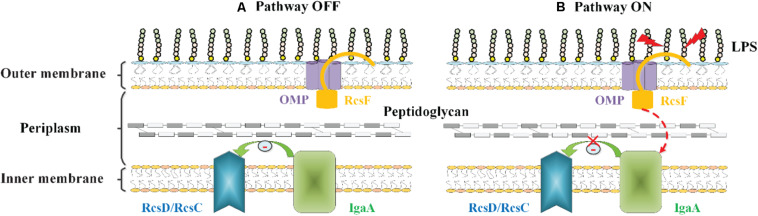
The role of RcsF/OMP complexes in sensing OM stress in *E. coli*. The OM lipoprotein RcsF is seated in an OMP. Its flexible, lipidated N-terminal domain is surface exposed, probing the state of LPS lateral interactions. The transmembrane segment of RcsF is threaded through the lumen of the OMP exposing the C-terminal domain in the periplasm. **(A)** In the absence of stress, IgaA inhibits the Rcs signaling, and the Rcs system is deactivated. **(B)** When LPS lateral interactions are disrupted by cationic antimicrobial peptides, or by the loss of negatively charged phosphate groups on the LPS molecule, this information is transduced to the RcsF C-terminal signaling domain located in the periplasm, resulting in the activation of the Rcs system by an unknown mechanism.

Konovalova et al. (2014, 2016) have proposed another model to explain the mechanism by which RcsF senses LPS defects. In their proposed model, RcsF forms a complex with the β-barrel and uses its positively charged, surface-exposed N-terminal domain to directly sense the state of LPS lateral interactions, thereby regulating the Rcs system activity (Konovalova et al., 2014, 2016). When LPS lateral interactions are perturbed by neutralization (by PMB or other cationic peptides), decreased LPS phosphorylation (biosynthesis defects), or a lack of cations to stabilize LPS cross-bridges (Mg^2+^ deficiency), this information is transduced to the RcsF C-terminal signaling domain located in the periplasm to activate the stress response (Konovalova et al., 2014, 2016) (Figure 4). However, it is not yet understood how the RcsF/OMP complexes transduce the induced signal from the cell surface to the IM component of the Rcs system.

To date, the RcsF-dependent signal transduction is not yet fully understood. Although two models have been proposed to explain the mechanism of RcsF activation, none of them have been confirmed. Even though, these findings clarify the mechanism by which RcsF acts as a “sentinel” in the Rcs signal transduction pathway that perceives and transmits signals, which is essential for analysis of the regulatory mechanism of the Rcs system.

#### RcsF-Independent Signal Transduction

Although RcsF is necessary for sensing most induction signals, not all Rcs input signals are RcsF-dependent. For example, overproduction of DjlA, a DnaJ homolog localized to the IM, was shown to activate the Rcs system in an *E. coli* strain with *rcsF* gene deletion (Shiba et al., 2004). Other RcsF-independent signaling pathways have been found in cell mutants of DsbA (Majdalani and Gottesman, 2005). DsbA is a periplasmic protein necessary for the formation of disulfide bonds, and this protein is vital for processes such as flagellar assembly (Heras et al., 2009). It has been shown that deletion of the *dsbA* gene can activate the Rcs system in *S. enterica*, and this activation is associated with the change in the disulfide bond state and therefore leads to incorrect assembly of the flagellar apparatus (Lin et al., 2008). It should be noted that these results occur only in RcsF mutants created in the laboratory and that no naturally occurring RcsF mutants have been observed. These results may reveal the cross-talk of the system when RcsF is not present. Cross-talk may be meaningful and this requires rigorous experimentation for clarification. The transcriptomics of wild type and *rcsF* mutant could be compared to screen signals or signaling proteins that crosstalk with the Rcs system. Although the Rcs system seems to function normally in the absence of RcsF, the mechanism by which the Rcs system senses signals independent of RcsF is still unknown and worth pursuing.

#### Virulence Regulation of the Rcs System in Several Common Pathogens

The Rcs system has an important regulatory function in bacterial virulence, mainly executed by regulating genes related to the bacterial surface structures (such as flagella, fimbriae, and extracellular polysaccharides). In addition, the Rcs system also regulates genes involved in the assembly of the secretion system and proteins that have predictive effects on cell surface maintenance and modification. Therefore, it is generally believed that the Rcs system affects bacterial motility, biofilm formation, intracellular survival, and invasiveness (Clarke, 2010; Guo and Sun, 2017; Wall et al., 2018). The Rcs system of different species exerts virulence regulation through different mechanisms, thereby affecting bacterial pathogenicity (Tobe et al., 2005; Erickson and Detweiler, 2006; Wang et al., 2009; Li Y. L. et al., 2015; Meng et al., 2020b).

#### Virulence Regulation of the Rcs System in *Salmonella*

The effect of the Rcs system on virulence regulation has been well studied in *S. enterica* serovar Typhimurium, a principal agent of gastroenteritis in humans (Detweiler et al., 2003; Mouslim et al., 2004; Garcia-Calderon et al., 2005). It has been reported that mutation in the *rcsC* allele (encoding a protein with constitutive kinase activity) can reduce the virulence of *S. enterica* in mice. This virulence attenuation depends on the RcsB phosphorylation level and partly depends on RcsA and colanic acid production (Mouslim et al., 2004; Garcia-Calderon et al., 2005). Additionally, a constitutively active RcsC (encoded by the *rcsC11* allele) was also shown to reduce the phagocytosis rates of *Salmonella* by murine macrophages, and this defect might be attributed to an increase in colanic acid production (Mouslim et al., 2004). It can be inferred that Rcs system overactivation in *Salmonella* is harmful to phagocytosis by macrophages and the persistent survival of bacteria in macrophages. Furthermore, the *Salmonella rcsC* gene was confirmed to play an important role in systemic infections in mice (Detweiler et al., 2003; Erickson and Detweiler, 2006). There is evidence that the *rcsC* mutant is less virulent to BALB/c mice than the wild-type strain. Further, after the mice were continuously infected by the *rcsC* mutant for 11 days, the recovery period of the spleen and liver of the mice was significantly lower than that of the wild-type strain (Detweiler et al., 2003). The *rcsC* gene in *S. enterica* serovar Typhimurium also regulates the expression of *ugd*, a gene required for the synthesis and incorporation of L-aminoarabinose into LPS to induce bacterial resistance to polymyxin B (Mouslim and Groisman, 2003).

Further studies show that the Rcs system is involved in the temporal regulation of virulence gene expression during *S. enterica* serovar Typhimurium infection. Studies have shown that the Rcs system in *Salmonella* can positively (low RcsB-P levels) or negatively (high RcsB-P levels) regulate the expression of the SPI-1 and SPI-2 pathogenicity island genes (Wang et al., 2007, 2009), which are important in the early stages of bacterial infection and can enable *S. enterica* to cross the epithelial barrier and enter macrophages (Lostroh and Lee, 2001; Fass and Groisman, 2009). In the early stages of infection, RcsB-P levels are low in the cells, which allow for the expression of genes related to motility, SPI-1, and SPI-2 pathogenicity islands. After *Salmonella* enters macrophages, environmental signals trigger the activation of RcsC kinase activity, leading to an increase in the level of RcsB-P, which in turn inhibits the expression of genes involved in motility, SPI-1, and SPI-2 pathogenicity islands. At this instance, the Rcs system regulates the expression of *ydeI* (encoding a 14-kDa periplasmic protein), which is important for persistent *S. enterica* serovar Typhimurium infection in mice (Erickson and Detweiler, 2006; Pilonieta et al., 2009). It can be inferred that even in the same bacteria, the Rcs system may have different regulatory effects on different stages of the same physiological process. A recent study showed that partially defective β-barrel assembly activated the RcsCDB regulon, leading to the decreased transcription of *hilA*, encoding the transcriptional activator of the SPI-1 structural genes (Palmer and Slauch, 2020). All these data indicate that expression of the SPI-1 pathogenicity island is tightly controlled in response to various regulatory inputs, and the Rcs system plays a vital role in this process.

#### Virulence Regulation of the Rcs System in *E. coli*

Enterohemorrhagic *E. coli* (EHEC) O157: H7 can cause hemorrhagic colitis with a low minimum infectious dose (Tobe et al., 2005). In this bacterium, the Rcs system in the inactive state negatively regulates expression of the locus of enterocyte effacement (LEE) pathogenicity island genes by inhibiting the *pch* regulatory gene, while the Rcs system in the active state positively regulates expression of the LEE pathogenicity island genes by inducing the expression of the *grvA* gene, thereby leading to enhanced expression of effector proteins in the type III secretion system (T3SS) (Tobe et al., 2005). The increase in these virulence factors leads to the enhanced adhesion and invasion of bacteria to the host cells, and finally promotes its infection ability (Tobe et al., 2005).

As the name indicates, the Rcs system is necessary for the synthesis of colanic acid capsules in *E. coli* K12 (Gottesman et al., 1985). Colanic acid was identified to be involved in some aspects of virulence in *E. coli*. For example, mutants in colanic acid production were identified in a signature-tagged mutagenesis (STM) study of avian pathogenic *E. coli*-mediated septicemia in chickens (Li et al., 2005). The recent screening of *E. coli* mutants that extend the lifespan of *Caenorhabditis elegans* revealed the role of colanic acid. It was found that overproduction of colanic acid could extend the lifespan of *C. elegans* and *Drosophila melanogaster* with *E. coli*, and that colanic acid itself demonstrated a similar effect (Han et al., 2017). Additionally, colanic acid is also important for biofilm formation in some strains of *E. coli* and for the optimal combination of *E. coli* O157:H7 with alfalfa sprouts (Danese et al., 2000; Matthysse et al., 2008).

The most studied effect of the Rcs system is its ability to inhibit bacterial motility and the expression of the *flhDC* operon, which encodes the master regulator of flagella production (Hagiwara et al., 2003; Mariscotti and Garcia-del Portillo, 2009; Wang et al., 2012; Howery et al., 2016). Flagella are considered as a surface organelle and assists in the initial attachment of bacteria, thus playing an important role in the development of *E. coli* biofilms (Houdt and Michiels, 2005). More importantly, flagellar motility is an important phenotypic characteristic of bacterial viability, competitiveness, and pathogenicity, and plays a key role in the early stage of *E. coli* infection (Josenhans and Suerbaum, 2002). In addition to *E. coli*, transcriptomic studies on bacteria such as *S. enterica*, *Erwinia amylovora*, and *Proteus mirabilis* have also determined the negative regulatory effect of the Rcs system on motility and *flhDC* expression (Mariscotti and Garcia-del Portillo, 2009; Wang et al., 2012; Howery et al., 2016). Similar to the system in *E. coli*, the Rcs system in these bacteria may affect cell motility, bacterial colonization, biofilm formation, and even the ability to infect the host by regulating flagella biosynthesis.

Studies have shown that the sRNA RprA in *E. coli* is positively regulated by the Rcs system, and this sRNA can base-pair with the 5′-end of *rpoS* mRNA (encoding selective σ factor, σ^s^), thereby increasing the translation level of the *rpoS* mRNA (Majdalani et al., 2002). Activation of the Rcs system can lead to an increase in the expression of RprA in *E. coli*, thereby negatively regulating biofilm formation (Majdalani et al., 2002). It has been reported that the biofilm formation defect in *E. coli* associated with *rcsC* or *rcsD* mutation is caused by the increased expression level of σ^s^ in the cells, and this defect can be restored by mutating *rcsB* or *rprA* (Ferrieres et al., 2009). A similar negative effect of Rcs activation on biofilm formation was also found in *S. enterica* serovar Typhimurium, which was also attributed to RprA and its ability to negatively regulate the master regulator for curli synthesis, CsgD (Latasa et al., 2012). Therefore, the Rcs system in *E. coli* seems to be a complex regulatory network involving gene regulation at the transcriptional and post-transcriptional levels.

#### Virulence Regulation of the Rcs System in *Yersinia*

The genus *Yersinia* belongs to the family *Enterobacteriaceae* and comprises 18 species of Gram-negative bacteria. The three documented species virulent to humans are: (i) *Yersinia pseudotuberculosis*, a zoonotic pathogen of mammals and birds that occasionally causes enterocolitis, mesenteric lymphadenitis, septicemia, and immune-mediated diseases in humans; (ii) *Yersinia pestis*, the causative agent of plague, including the medieval “Black Death”; and (iii) *Y. enterocolitica*, the *Yersinia* species most frequently associated with human infections. The Rcs system has been proven to regulate the virulence of the three bacteria (Fang et al., 2015; Li Y. L. et al., 2015; Meng et al., 2020b).

In *Y. pseudotuberculosis*, the Rcs system can positively regulate Ysc-Yop T3SS by regulating transcription of the *virG-lcrF* operon (encoding LcrF), which increases the Yop effector protein secreted by the bacteria in the host immune cells, thereby enhancing survival of the bacteria (Li Y. L. et al., 2015). Additionally, a null mutation in the *rcsD* gene of *Y. pseudotuberculosis* results in decreased adhesion to epithelial cells, an important prerequisite to infection (Hinchliffe et al., 2008).

The formation of biofilms can enhance the transmission of the plague pathogen *Y. pestis* in the midgut of its flea host (Sun et al., 2011). In *Y. pestis*, the Rcs system was identified to regulate the environmental adaptation of *Y. pestis* by regulating production of c-di-GMP and synthesis of extracellular polysaccharides in the biofilm matrix (Fang et al., 2015).

In *Y. enterocolitica*, *rcsB* deletion significantly downregulated the expression of Ysa T3SS, which is involved in the colonization of *Y. enterocolitica* in the terminal ileum of mice. Studies show that the *rcsB* mutant has a disadvantage in fitness when co-infected in mice with wild-type *Y. enterocolitica* (Venecia and Young, 2005; Walker and Miller, 2009). Recent studies in our laboratory have shown that the lack of *rcsB* activates the adhesion and invasion ability of *Y. enterocolitica* to Caco-2 cells, which may be attributed to the activation of flagella synthesis and bacterial chemotaxis by the *rcsB* mutation (Meng et al., 2020b). Noteworthy, the regulation of *Y. enterocolitica* infection by the Rcs system was distinctly different *in vivo* and *in vitro*. Furthermore, transcriptomic analysis showed that loss of *Y. enterocolitica rcsB* resulted in significant downregulation of *phoQ* and *pagP* (Meng et al., 2020b). *pagP* is the target gene of the PhoP/PhoQ TCS and is responsible for the modification of the LPS structure for improvement of the resistance of bacteria to polymyxin B (Wang et al., 2009). Therefore, combined with the positive effect of RcsB on resistance to polymyxin B as previously determined (Meng et al., 2019), this evidence led us to suggest that the Rcs system positively regulated the activity of the PhoP/PhoQ system to equip *Y. enterocolitica* with a certain level of resistance to polymyxin B.

#### Virulence Regulation of the Rcs System in Other Members of the *Enterobacteriaceae* Family

In *Klebsiella pneumonia*, the Rcs system would regulate the expression of capsule polysaccharide (CPS) by regulating the CPS-related genes; further, the Rcs system in *Klebsiella pneumoniae* repressed the expression of *fim* gene cluster, which could acts as a virulence factor that facilitates the urinary tract infection (Su et al., 2018). Additionally, as found in *Y. enterocolitica*, there is also a cross talk between PhoP/PhoQ system and Rcs system in regulating the resistance of bacteria to antimicrobial peptides (Llobet et al., 2011). The Rcs system was also identified as a regulator of two well-studied virulence genes, namely *zapA*, encoding a type-1 secretion ATP-binding protein, and *hpmBA* encoding hemolysin in *P. mirabilis* (Howery et al., 2016). Additionally, the Rcs system in *P. mirabilis* repressed the expression of *mrpA*, *pmfA*, and *ucaA* genes involved in the formation of fimbriae, a cell surface structure that may affect the ability of bacteria to form biofilms and infect the host (Howery et al., 2016). In *Serratia marcescens*, RcsB inhibits the pore-forming toxin ShlA, which is responsible for the early induction of autophagy in host cells (Di Venanzio et al., 2014). The Rcs system has been reported to be essential for *Er. amylovora* virulence by controlling amylovoran biosynthesis, which is one of the main pathogenic factors of this bacterium that is exhibited as ooze in infected tissues (Wang et al., 2009, 2012). Notably, *Er. amylovora* RcsC positively controls the expression of amylovoran biosynthetic genes *in vivo* but negatively controls their expression *in vitro* (Wang et al., 2012), which further proves that the Rcs system exhibits differences in the regulation of gene expression *in vivo* and *in vitro*.

## Conclusion and Future Perspectives

The Rcs system is an important signal transduction pathway found in many members of the *Enterobacteriaceae* family. This system can integrate environmental signals, regulate gene expression, and alter the physiological behavior of bacteria. The OM protein RcsF can sense envelope stress signals that activate the Rcs system, trigger the downstream signal transmission of the Rcs system in the order RcsC→RcsD→RcsB, and finally regulate the transcription of target genes. The dual function of RcsC, the phosphorylation modification of RcsB, and the participation of multiple auxiliary proteins lead to the complexity and flexibility of the Rcs system, thereby achieving precise regulation of its target genes. Additionally, the Rcs system can exert its virulence regulation function through different mechanisms, thereby affecting the pathogenicity of bacteria. This review summarizes the role of the Rcs system in ESRs and virulence regulation in different pathogens. It can be inferred that the Rcs system plays an important role in the environmental response of bacteria. However, there are many relevant issues that need to be addressed urgently. For example, (i) in the absence of RcsF, the Rcs system continues to function normally. For RcsF-independent signals, the mechanism by which the Rcs system senses signal molecules and achieves signal transduction remains unknown; (ii) to date, most studies have identified Rcs-regulated genes under *in vitro* growth conditions or by over-activating the Rcs system, and few studies have performed genome-wide gene expression under *in vivo* conditions. Owing to the differences in the regulation of gene expression by the Rcs system *in vivo* and *in vitro*, it is necessary to conduct transcriptomic studies of the Rcs system *in vivo* to identify new virulence-related target genes. In summary, the Rcs system provides a unique model for studying the complexity of environmental adaptation in bacteria. An understanding of the Rcs system will help to provide a theoretical basis for the development of control, prevention, and treatment of bacterial infections.

## Author Contributions

JM, GY, and JC wrote the manuscript. All authors have approved the final version of the manuscript for publication.

## Conflict of Interest

The authors declare that the research was conducted in the absence of any commercial or financial relationships that could be construed as a potential conflict of interest.
